# Quantifying Efficacy and Limits of Unmanned Aerial Vehicle (UAV) Technology for Weed Seedling Detection as Affected by Sensor Resolution

**DOI:** 10.3390/s150305609

**Published:** 2015-03-06

**Authors:** José M. Peña, Jorge Torres-Sánchez, Angélica Serrano-Pérez, Ana I. de Castro, Francisca López-Granados

**Affiliations:** Institute for Sustainable Agriculture, IAS-CSIC, P.O. Box 4084, 14080 Córdoba, Spain; E-Mails: jtorres@ias.csic.es (J.T.-S.); aserrano@ias.csic.es (A.S.-P.); anadecastro@ias.csic.es (A.I.C.); flgranados@ias.csic.es (F.L.-G.)

**Keywords:** remote sensing, visible-light and multispectral cameras, object-based image analysis (OBIA), weed mapping, site-specific weed management (SSWM)

## Abstract

In order to optimize the application of herbicides in weed-crop systems, accurate and timely weed maps of the crop-field are required. In this context, this investigation quantified the efficacy and limitations of remote images collected with an unmanned aerial vehicle (UAV) for early detection of weed seedlings. The ability to discriminate weeds was significantly affected by the imagery spectral (type of camera), spatial (flight altitude) and temporal (the date of the study) resolutions. The colour-infrared images captured at 40 m and 50 days after sowing (date 2), when plants had 5–6 true leaves, had the highest weed detection accuracy (up to 91%). At this flight altitude, the images captured before date 2 had slightly better results than the images captured later. However, this trend changed in the visible-light images captured at 60 m and higher, which had notably better results on date 3 (57 days after sowing) because of the larger size of the weed plants. Our results showed the requirements on spectral and spatial resolutions needed to generate a suitable weed map early in the growing season, as well as the best moment for the UAV image acquisition, with the ultimate objective of applying site-specific weed management operations.

## 1. Introduction

Sunflower is the most important annual oilseed crop in southern Europe and the Black Sea area, with over 5 M·ha grown annually [1]. Spain has 0.8 M·ha of sunflowers [2]. The patchy distribution of weeds in sunflower fields has already been demonstrated using on-ground sampling [3,4] and remote imagery from piloted aircraft [5]. Although the distribution of weeds is patchy, herbicides are usually broadcast over entire fields, even onto the weed-free areas. To overcome this problem, site-specific weed management (SSWM) is used to spray an adapted herbicide treatment only on weed patches and/or to adjust different herbicide applications according to weed species composition, e.g., herbicide resistant, broadleaved or grass weeds. Thus, one of the crucial components for SSWM is accurate and timely weed maps, which must be generated to design the corresponding site-specific herbicide applications [6]. With the SSWM approach, the hope is to also reduce herbicide use.

This strategy fits well with European concerns on herbicide use (Horizon 2020, European Commission, Societal Challenge 2: Sustainable Food Security. SFS-3-2014: Practical solutions for native and alien pests—including weeds—affecting crops) and has prompted the European Union to enact restrictive legislation (Regulation EC No. 1107/2009 and Directive 2009/128/EC). The legislation requires action to achieve the sustainable use of pesticides and to promote the use of the most advanced and latest technologies. Of the advanced technologies in weed research today, one of the most promising and innovative is the use of Unmanned Aerial Vehicles (UAVs or drones) equipped with a perception system for mapping weeds. The maps generated from the remote images captured with the UAV can be used for the further design of appropriate site-specific control measures.

Compared with other remote platforms such as satellites or piloted aircrafts, UAVs can operate at low altitudes (e.g., <120 m), even on cloudy days, and can provide an ultra-high spatial resolution (e.g., pixels < 3 cm) image of the entire crop field. Configurations and specifications for an UAV to map weeds for early site-specific weed management have been reported by [7]. The UAV can be programmed on demand and it can fly with great flexibility and collect remote imagery of crops at critical times in the growing season, thereby improving the farmer’s decision-making process [8]. The total availability is fundamental for UAVs to perform a multi-temporal study in early weed detection and to determine the best time for taking the imagery needed to design post-emergence herbicide control strategies, just when the crop and weeds have similar appearance and spectral characteristics [9,10]. With the high spatial and temporal resolution requirements and the spectral similarity between weed and crop seedlings, remote-sensed discrimination of early-season crop and weeds remains a challenge in weed research.

According to [11], one of the inherent problems with increasing the spatial resolution of remote images is that single pixels no longer capture the characteristics of classification targets. This produces an increase in intra-class spectral variability and, subsequently, a reduction in statistical separability among classes with conventional pixel-based classification methods, which can involve a reduction in classification performance and accuracy in comparison with coarser resolution images. Object-based image analysis (OBIA) is a powerful procedure and a fine alternative to the pixel-based methods [12]. The OBIA approach first identifies spatially and spectrally homogenous units (objects) created by grouping adjacent pixels according to a procedure known as segmentation. It then develops automated and auto-adaptive classification methods by using the objects as the minimum information units and combining their spectral, contextual (position, orientation), morphological and hierarchical information. This methodology has been used successfully for segmenting and classifying a QuickBird satellite image as the first step in isolating wheat fields from other soil uses for further detection of cruciferous weed patches at a late growth stage [13]. Recently, [14] translated the OBIA strategy to early-season weed discrimination in maize by using UAV imagery and a three-step automatic classification approach: (1) image segmentation into multi-pixel regions that define plants (crop and weeds) and soil background objects; (2) discrimination of vegetation objects based on spectral information; and (3) classification of crop and weed plants based on the position of each plant relative to the crop rows. This OBIA strategy produced maps of three weed coverage categories, and [14] concluded that an accurate definition of the crop-row structure was essential for the subsequent discrimination between crop and weeds.

Another crucial point for improving the discrimination of weeds in ultra-high spatial resolution images would be to enhance the differences among vegetation and non-vegetation (mostly bare soil) objects by using vegetation indices as well as to determine the optimal threshold value that sets the breakpoint between both general classes [15]. One of the automatic methods for threshold calculation is Otsu’s [16], which is commonly applied to binary classification (in our case, bare soil and vegetation) and calculates the optimum threshold based on minimising combined spread (intra-class variance). A recent evaluation of the performance of Otsu’s threshold method in UAV images [10,17] considered two different vegetation indices as well as the influence of image resolution and objects size (*i.e.*, segmentation scale), and concluded that these parameters are critical to accurately characterise the spectral threshold for a precise classification of vegetation (crop and weeds) and non-vegetation objects.

Accounting for the factors introduced previously, the objectives of this work were as follows: (1) to determine the optimum configuration of the UAV flight for the altitude, the date of flight (*i.e.*, crop and weed phenological stage) and the type of sensor (visible-light *vs.* visible-light + near-infrared multispectral cameras); (2) to determine the best sensor for enhancing vegetation (crop and weed) and bare soil class discrimination as affected by the vegetation index applied; and (3) to design and evaluate an OBIA procedure for crop and weed patch detection. Limitations and opportunities of using higher flight altitudes were also analysed for each sensor, aiming to optimise the image acquisition and classification processes.

## 2. Experimental Section

### 2.1. Study Site

The multi-temporal study was carried out in a sunflower field situated at the public farm Alameda del Obispo, in Córdoba (southern Spain, coordinates 37,856N, 4806W, datum WGS84). The sunflower crop was sown on 15 April 2014, at 6 kg·ha^−1^ in rows 0.70 m apart, and emergence of the sunflower plants began 15 days after sowing (DAS). An area of approximately 0.5 ha, with flat ground (average slope <1%) and naturally infested by broadleaved weeds such as *Chenopodium album* L. and *Convolvulus arvensis* L, was studied in detail. Weed and crop plants were in the principal stage 1 (leaf development) from the BBCH extended scale [18] during the study and grew from four true leaves (code 14–16) in the beginning of the experiment to eight true leaves (code 18) at the end.

### 2.2. UAV Flights: Camera, Altitudes and Dates

**Figure 1:**
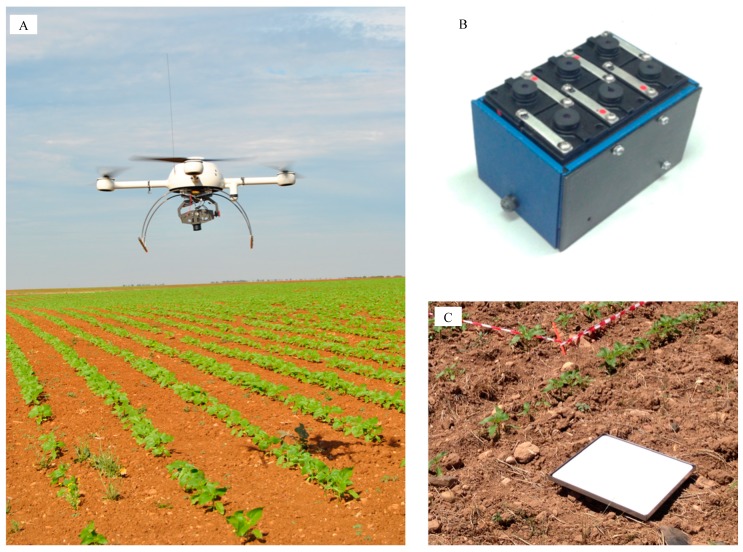
(**A**) Unmanned aerial vehicle (UAV), model microdrone MD4-1000, with the visible-light camera attached, flying over the sunflower crop in the early season; (**B**) TetraCam Multispectral camera; and (**C**) Spectralon^®^ panel placed in the middle of the field to calibrate the spectral data.

The remote images were acquired with two different cameras mounted separately in a quadrocopter UAV, model md4-1000 (microdrones GmbH, Siegen, Germany, Figure 1A): (1) a conventional still visible-light camera, model Olympus PEN E-PM1 (Olympus Corporation, Tokyo, Japan), which acquired 12-megapixel images in true Red-Green-Blue (RGB) colour with 8-bit radiometric resolution; and (2) a multispectral camera, model Tetracam mini-MCA-6 (Tetracam Inc., Chatsworth, CA, USA), which acquired 1.3-megapixel images composed of six individual digital channels arranged in a 2 × 3 array that can acquire images with either 8-bit or 10-bit radiometric resolution (Figure 1B). This camera has user configurable band pass filters (Andover Corporation, Salem, NH, USA) of 10-nm full-width at half maximum and centre wavelengths at B (450 nm), G (530 nm), R (670 and 700 nm), R edge (740 nm) and near-infrared (NIR, 780 nm).

**Figure 2:**
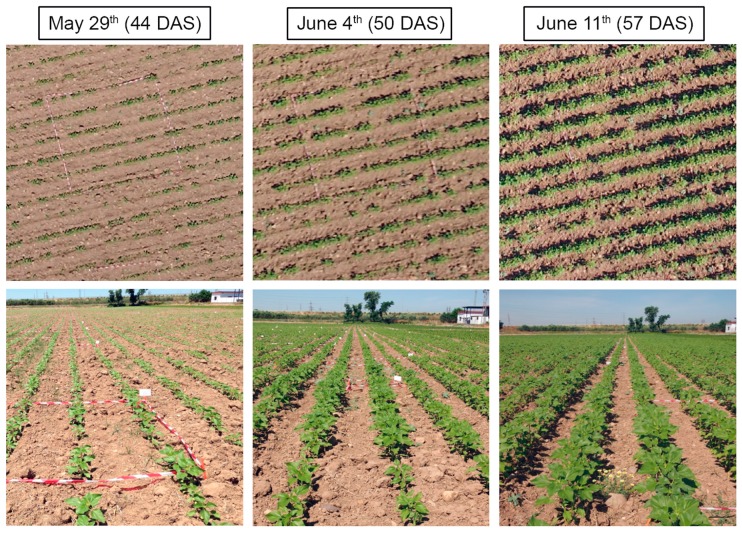
UAV images collected over the sunflower field at 40 m on three different dates in the early season (**top**) and associated on-ground photograph (**bottom**).

Detailed information on the configuration of the UAV flights and specifications of the vehicle and the cameras can be found in [7]. A set of aerial images was collected at intervals of 6–7 days on 29 May (date 1, 44 DAS), 4 June (date 2, 50 DAS) and 11 June (date 3, 57 DAS) to quantify multi-temporal discrimination of weeds and crop at the different growth stages described previously (Figure 2). On each date, flights for each camera were conducted at four different altitudes: 40, 60, 80 and 100 m. Each flight route was programmed into the UAV software so that the vehicle ascended vertically above a fixed point in the sunflower field. Once the UAV achieved each programmed altitude, a unique image was captured as the vehicle stopped. In total, twenty four images were taken and analysed, which were geo-referenced by identifying a set of ground target points located in the field by using a GPS and attributing their coordinates to the remote images by using the ENVI software (ENVI 4.4., Research Systems Inc., Boulder, CO, USA).

In the course of the UAV flights, a barium sulphate standard Spectralon^®^ panel (Labsphere Inc., North Sutton, NH, USA) of 0.45 × 0.45 m (Figure 1C) was placed in the middle of the field to correct the image data for the effects of shifting light conditions (e.g., due to changes in solar elevation or clouds) over time (several flight missions in three different dates). Digital images were spectrally corrected by applying an empirical linear relationship in which the equation coefficients were derived by fitting the digital numbers of the image pixels located in the Spectralon panel to the Spectralon ground values [19]. The images taken with the visible-light camera were used directly after downloading to the computer, but images taken with the multispectral camera required preprocessing. This camera takes the images of each channel in raw format and stores them separately on six individual CF cards embedded in the camera. Therefore, an alignment process was needed to group the six single images into a multi-band image. The Tetracam PixelWrench 2 software (Tetracam Inc.) supplied with the multispectral camera was used to perform the alignment process.

### 2.3. OBIA Algorithm

The OBIA procedure designed for the weed mapping tasks was developed using the commercial software eCognition Developer 8.9 (Trimble GeoSpatial, Munich, Germany). It was based on the weed mapping algorithm fully described in our previous work conducted in early-season maize fields [14,20]. However, the procedure presented here is original and includes improvements and variations related to the special characteristics of sunflower crops.

**Figure 3:**
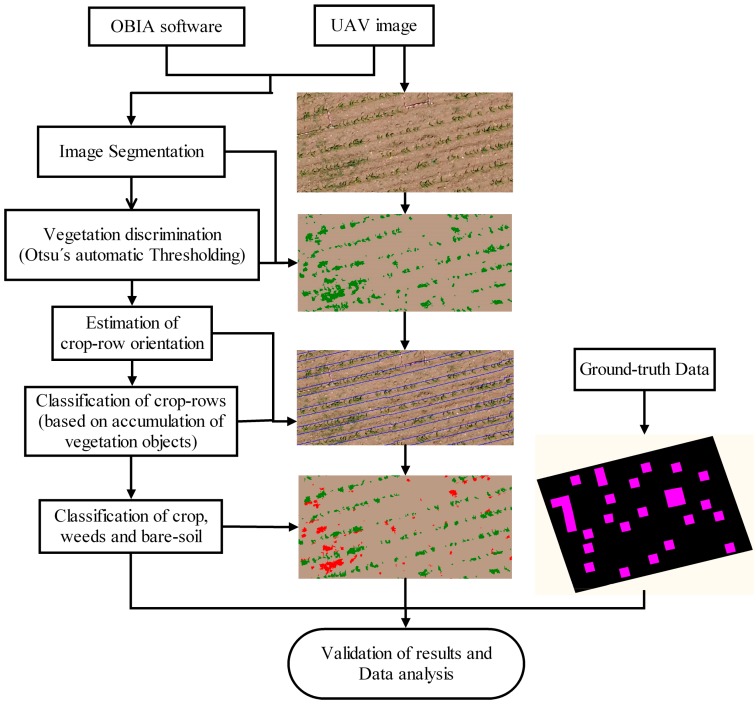
Flowchart of the OBIA procedure applied for crop-row classification and weed detection.

The OBIA algorithm combined object-based features such as spectral values, position, orientation and hierarchical relationships among analysis levels; the algorithm recognised that the plants growing on the surface between crop rows were weed plants. Therefore, the algorithm was programmed to accurately detect the crop rows by the application of a dynamic and auto-adaptive classification process, and then classified the vegetation objects outside the crop rows as weeds. The flowchart of the detailed image analysis can be examined in [14]; in this paper, only the main variations and upgrades are emphasised. The entire process is automatic and is composed of a sequence of routines described as follows (Figure 3):

(a) Field segmentation in sub-plots: The algorithm segmented the UAV images into small plots of a customised size to address the spatial and spectral variability of the crop field. In our case, sub-plots of 5 × 5 m were selected and sequentially analysed.
(b) Sub-plots segmentation in objects: The image sub-plots were sub-segmented using the multi-resolution algorithm implemented in eCognition to create multi-pixel objects representing the elements of the fields, *i.e.*, crop and weed plants (vegetation objects) and soil background (bare soil objects). Segmentation is a bottom-up region-merging process based on band weights and on five parameters (scale, colour, shape, smoothness and compactness) defined by the operator. After visually testing several segmentation outputs, the selected values were 10, 0.9, 0.1, 0.5 and 0.5 for scale, colour, shape, smoothness and compactness, respectively. Within the range of spatial resolutions (a few centimetres) studied in this investigation, this segmentation setting was adequate for all the studied scenarios. However, this issue merits further investigation aiming to optimize the segmentation setting as affected by the crop pattern (e.g., crop row separation) and image spatial resolution [17]. The resulting objects contained new contextual and morphological information (e.g., orientation, position, size, shape, and others) that were used in the next phases of the classification process.
(c) Vegetation objects discrimination: After segmentation, the first step in the classification process was to discriminate the vegetation objects from the bare soil objects. Two spectral indices were used: (1) the Excess Green index (ExG, Equation (1)) for the visible-light camera [21,22]; and (2) the Normalised Difference Vegetation Index (NDVI, Equation (2)) for the multispectral camera [23]. The indices were calculated as follows:
  (1) $$ExG=2g-r-b;\;r=\frac{R}{R+G+B}\;g=\frac{G}{R+G+B}\;b=\frac{B}{R+G+B}$$
  (2) $$NDVI=\frac{NIR-R\;}{NIR+R}$$

These indices enhance spectral differences of vegetation objects against the non-vegetation ones as previously reported by [7], while minimizing solar radiance and soil background effects [24]. The determination of the optimum ExG and NDVI values for vegetation discrimination in the UAV images was conducted by an automatic and iterative threshold approach following the method of Otsu [16] and implemented in eCognition according to [17]:

(d) Crop-row classification: Once the vegetation objects were discriminated, the crop-row structure was classified by following three steps: (1) estimation of the crop-row orientation; (2) image gridding based on stripes following the crop-row orientation; and (3) crop-row classification. First, crop-row orientation was determined by an iterative process in which the image was repeatedly segmented in stripes with different angles (from 0° to 180°, with 1° of increase ratio), with the selected orientation the one in which the stripes showed a higher percentage of vegetation objects. Next, a new segmentation level (*i.e.*, upper level) was created above the previous multi-resolution one (*i.e.*, lower level) in which the image was segmented to create a mesh of stripes with the same direction as the selected crop-row orientation angle. Finally, the stripe in the upper segmentation level with the higher percentage of vegetation objects in the lower segmentation level were classified as crop rows, following the criteria described in [25]. In this process, after a stripe was classified as a crop-row, the separation distance between rows (0.7 m in sunflower) was used to mask the neighbouring stripes within this distance, which avoided classifying areas with high weed infestation as crop rows.
(e) Weed and crop discrimination: Once the crop-rows were classified, the remaining stripes were classified as crop-row buffer (strings in contact with the crop rows) and non-crop area in the upper segmentation level. Next, the hierarchical relationship between the upper and the lower segmentation levels was used to execute the discrimination of crop and weeds. The vegetation objects (in the lower segmentation level) that were located either under the crop rows or under the non-crop area (in the upper segmentation level) were classified either as sunflower or as weeds, respectively. The remaining vegetation objects located under the buffer area were classified following a criterion of minimum spectral distance, *i.e.*, an unclassified vegetation object was assigned to the sunflower or weed class depending on its higher degree of spectral similarity to its surrounding sunflower or weed objects, respectively.
(f) Weed coverage assessment: A vector file containing 30 geo-referenced sampling frames, 1 × 1 m in size, was overlapped in the classified image to calculate the relative area corresponding to each class, *i.e.*, sunflower, weeds and bare soil, in every frame. Weed coverage was determined as the percentage of pixels classified as weed per unit of ground surface. Information derived from these frames was used for validation purposes, as explained in the next section.

### 2.4. Evaluation of OBIA Algorithm Performance

**Figure 4:**
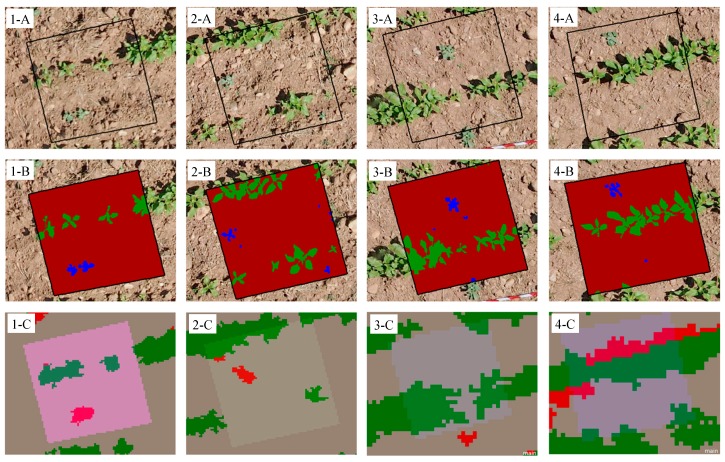
Examples of four sampling frames showing: (**1**) correct classification; (**2**) underestimation of weeds; (**3**) false negative errors (*i.e.*, no detection of weeds); and (**4**) false positive errors (*i.e.*, overestimation of weeds) in three scenarios: (**A**) On-ground photographs; (**B**) manual classification of observed data; and (**C**) image classification performed by the OBIA algorithm.

The performance of the OBIA algorithm in each case study (each camera, flight altitude and date) was evaluated by visually comparing the results obtained for crop-row identification and weed discrimination with real data observed in 30 ground-truth 1 × 1 m^2^ sampling frames in the field. These sampling areas were regularly distributed in the study area and were representative of the weed infestation observed in the field and included a number of sampling frames free of weeds. Ground-truth observations were derived from vertical remote images collected with a UAV flight at 10 m. For this purpose, the UAV equipped with the visible-light camera was programmed to fly continuously taking overlapped images every second (80% forward-lap and 30% side-lap). The set of UAV images were mosaicked using Agisoft Photoscan Professional Edition (Agisoft LLC, St. Petersburg, Russia) software following the protocol described in [7,26]. Because of the low flight altitude, the mosaicked image had 0.38 cm/pixel of spatial resolution, which made it possible to visually identify the individual plants in every reference frame and thus conduct a manual classification of the ground-truth data for crop plants, weeds and bare-soil (Figure 4). By comparing observed data and classification outputs in each case study, the OBIA algorithm was evaluated by quantifying the number of correct frames, *i.e.*, those sampling frames in which all the weed plants were correctly attributed to weed objects (Figure 4(1-C)). Alternatively, incorrect frames (e.g., crop plants classified as weed objects, weed plants classified as bare soil objects, *etc.*) were also labelled as three different types: (1) underestimated, *i.e.*, weed-infested frames in which some weed plants were detected but other weed plants remained undetected by the OBIA algorithm (Figure 4(2-C)); (2) false negative, *i.e.*, weed-infested frames in which no weeds were detected (Figure 4(3-C)); and (3) false positive, *i.e.*, frames in which weeds were overestimated (e.g., crop plants or bare soil elements classified as weed objects) (Figure 4(4-C)).

## 3. Results and Discussion

### 3.1. Image Spatial Resolution and Covered Area As Affected by Flight Altitude

**Figure 5:**
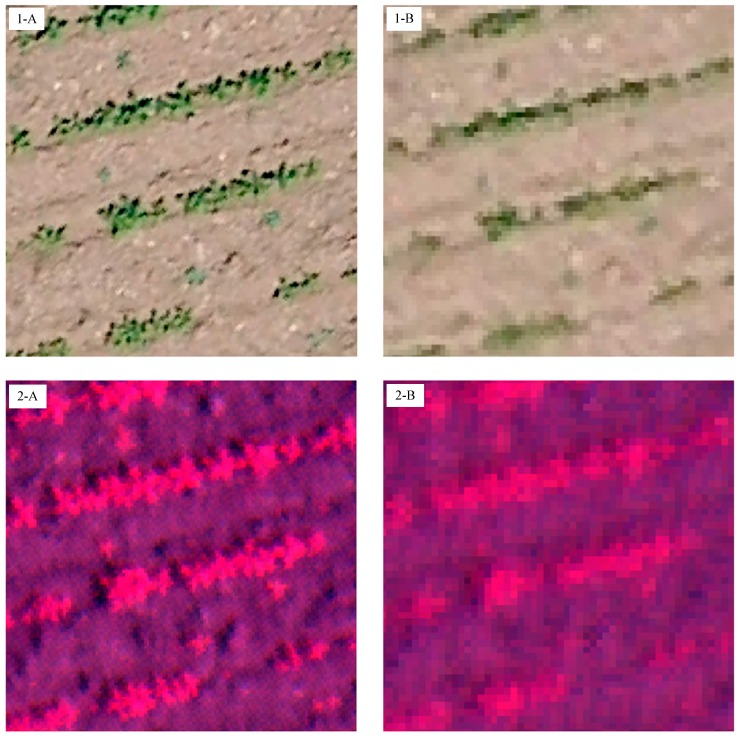
Details of the image spatial resolution captured by the visible-light camera (1) and the multispectral camera (2) at: (**A**) 40 m altitude; and (**B**) 100 m altitude.

The image spatial resolution captured by each camera and the area covered by each individual image at different UAV flight altitudes are shown in Table 1. The visible-light and the multispectral cameras captured images with pixel sizes ranging from 1.52 cm to 3.81 cm and from 2.16 cm to 5.41 cm at flight altitudes of 40 and 100 m, respectively (Figure 5), as determined by a proportional relationship between sensor resolution and flight altitude. The ultra-high spatial resolution of the sensors is one of the crucial features for weed mapping early in the season when crop and weeds are at a young phenological stage (e.g., four true leaves).

**Table 1:** Image spatial resolution (pixel size) and area covered as affected by flight altitude and type of camera.

Flight Altitude	Pixel Size (cm)		Covered Area (ha)	
	Visible-Light Camera	Multispectral Camera	Visible-Light Camera	Multispectral Camera
40 m	1.52	2.16	0.28	0.06
60 m	2.28	3.27	0.63	0.14
80 m	3.04	4.33	1.13	0.25
100 m	3.81	5.41	1.77	0.38

**Figure 6:**
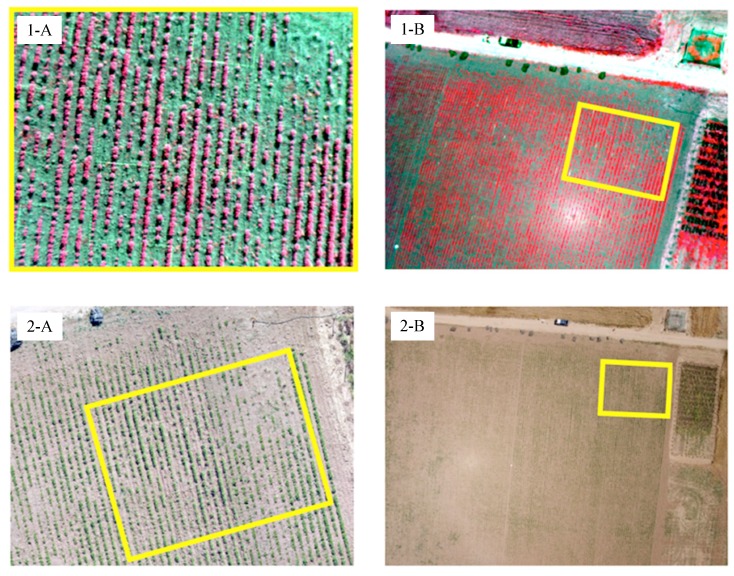
Individual UAV images collected with the multispectral (**1**) and the visible-light (**2**) cameras at: 40 m (**A**); and 100 m (**B**) altitude. The yellow squares serve to compare the area covered by the images from each camera at both flight altitudes. The sunlight effect (light spot) observed in 1-B and 2-B were minimized after applying vegetation indices (see Section 2.3).

In general, at least four pixels are required to detect the smallest objects within an image [27]. Accordingly, if the discrimination of individual weed plants is the objective, the pixel size should be approximately 1–4 cm, which corresponds to flight altitudes of 40 to 100 m for the visible-light camera and altitudes of 40 to 60 m for the multispectral camera. However, if the objective is weed patch detection, the pixel size of remote images could be 5 cm or even greater, which corresponds to a flight altitude of 100 m or higher for both cameras. One of the most relevant parameters was the area overlap because of its strong implications for the configuration of the optimum flight mission. This parameter is directly related to the flight altitude and the type of camera. At the flight altitudes in this study, each remote image captured with the visible-light camera covered approximately 4.6 times more surface area than the multispectral camera, e.g., increasing from 0.06 ha to 0.28 ha at 40 m and from 0.38 to 1.77 ha at 100 m, respectively (Figure 6).

The differences in pixel size and covered area were because of the technical specifications of each camera, since the camera focal length affects both parameters, whereas the camera sensor size only affects the image’s pixel size. Accordingly, when the user defines the flight program, it is necessary to balance the flight project to keep the image spatial and spectral quality steady, as the area covered is considered. Two main conditions must accounted for: (1) to provide remote images with a fine enough spatial and spectral resolution to guarantee weed discrimination at early growth stages; and (2) to cover as much surface area as possible to optimise the operation length of the UAV flight.

### 3.2. Accuracy Assessment on Classification of Crop-Rows

**Figure 7:**
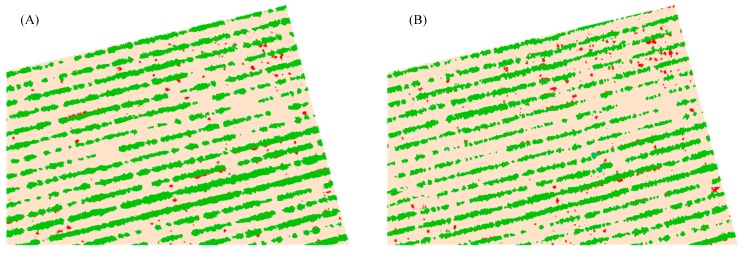
Image classified in weeds (red), sunflower crop rows (green) and bare soil (brown) using an UAV flying at 40 m altitude with: (**A**) visible-light camera (ExG index); and (**B**) multispectral camera (NDVI index).

The OBIA algorithm identified and counted the number of sunflower rows with 100% accuracy in all the images, independent of the camera type, date or flight altitude (Figure 7). This demonstrated the efficiency and robustness of the procedure developed for crop-row classification in which the stripes with the higher percentage of vegetation objects were selected as seeds for crop-row identification. This has strong implications for the success of the next steps in the OBIA procedure designed for weed discrimination, which should be focused on minimising potential errors in detecting the vegetation objects (weeds) located in the area between the sunflower rows.

### 3.3. Weed Discrimination As Affected by Camera, Date and Flight Altitude

Accuracy assessment on weed discrimination attained from analyses of the UAV images captured by each camera on each flight (four flight altitudes and three flight dates) is shown in Table 2. A ground-truth frame was classified as correct if all the weed plants within the frame were correctly attributed to weed objects. Otherwise, the frame was labelled as either underestimated, false negative or false positive according to the error observed (see Section 2.4). On the first date (44 DAS, when crop and weed were at the four true leaf phenological stage), 71% classification accuracy for both cameras was obtained for discrimination of weeds at the lowest altitude (40 m). However, at higher flight altitudes, the multispectral camera had higher accuracy (from 62% at 60 m to 43% at 100 m) than the visible-light camera (43% and 19%, respectively). From the analysis of the errors, most errors at 40, 60 and 80 m were attributed to false-negative errors, *i.e.*, misclassification was produced by non-detection of weeds (Figure 4(3-C)) or, of minor importance, because of the underestimation of weed coverage (Figure 4(2-C)). At 100 m, the trend was maintained in images captured with the multispectral camera but not with the images captured by the visible-light camera because, in the latter case, most of the errors were due to false positives (47%), which was attributed to classification of sunflowers as weeds (Figure 4(4-C)). This source of error gains importance at higher altitudes because of the spectral mixture between sunflowers and bare soil elements that occurred in the edges of the crop-rows. Because of a loss of spatial resolution, spectral information of the row edge pixels is a mix of sunflower (high ExG and NDVI values) and bare soil (low ExG and NDVI values) spectral response, which is similar to the weed spectral response in some cases (mainly in the visible-light images) and, as a result, it causes over-classification of weeds in the crop-row edges. On the first date, the algorithm for weed detection performed better for imagery captured with the multispectral camera at any of the flight altitudes even with its lower spatial resolution, compared with the visible-light imagery. This indicated that the near-infrared spectral information used by the multispectral camera is more suitable for this task and compensated for its lower spatial resolution (Table 1). From an agronomic point of view, the false positive errors (overestimation of weeds) are more acceptable than false negative errors (non-detection of weeds) for generating the weed maps in practice, assuming that farmers would choose to treat weed-free areas rather than risk allowing weeds to go untreated [28]. However, both cameras were fully effective (100% accuracy) at 40 and 60 m in the classification of the weed-free areas, which drastically reduced the potential impact of an overestimation of the weeds at the field scale. The accuracy was maintained by the images captured at 80 and 100 m with the multispectral camera but not with the visible-light images (44% and 33% accuracy, respectively).

On the second date (50 DAS, when crop and weeds were at the five-six true leaf phenological stage), the weed detection procedure found higher classification accuracy for both cameras, resulting in 77% and 91% of correct weed-infested frames at 40 m for the visible-light and the multispectral cameras, respectively. Similar to results from the previous date, the classification accuracy decreased in all the images with increasing altitude. The majority of errors were attributed to no-detection (false negative) and underestimation of weeds, although the highest value was the false positive error (41%) from the visible-light images captured at 100 m because of incorrect classification of the crop-row edges as weeds (Figure 4(4-C)). In the weed-free frames, the images captured with the multispectral camera were 100% accurate at 40, 60 and 80 m, although the OBIA algorithm slightly decreased its accuracy to 88% at 100. In the visible-light images, the results followed the trend of the first date, although the images had lower accuracy at 40 and 60 m (88%) and higher accuracy at 80 m (63%) and 100 m (37%) in comparison with the previous date. As an example, the weed maps generated on the second date from the images captured by both sensors at 40 m (best scenario) are shown in the Figure 7. Weed coverage was found to be 1.25% of the field area with the NDVI images (multispectral camera) and 0.98% with the ExG images (visible-light camera).

On the third date (57 DAS, when crop and weeds were at the seven-eight true leaf phenological stage), lower accuracy of weed detection was found, in general, than on the previous dates for the majority of the images and flight altitudes analysed, with the exception of the visible-light images captured at 60 and 80 m. For example, the accuracy of weed detection in the images captured at 40 m was 3% and 9% lower with the visible-light camera (ExG images) and 11% and 31% lower with the multispectral camera (NDVI images) in comparison with the results from date 1 and 2, respectively. On this date (57 DAS), the highest percentage of errors was mainly from non-detection of weeds (false negative) in both types of images, although primarily in the images captured with the multispectral camera. Although the weed plants were bigger on this date and their discrimination supposedly easier, weeds were masked by sunflower shadows, which increased the degree of weed misclassification. False positive errors were also an important source of confusion in the visible-light images captured at 80 and 100 m, occurring in 36% and 32% of the weed-infested frames, respectively, and in 37% of the weed-free frames at both altitudes. As on the previous dates, performance of the OBIA algorithm in the weed-free zones was very satisfactory with the multispectral camera at any altitude and with the visible-light camera at 40 and 60 m.

After the importance of efficient classification of crop-rows, spectral information derived from ExG (in the visible-light images) and NDVI (in the images captured with the multispectral camera) indices could initially be considered the primary factor affecting weed identification. However, image spatial resolution (or, equally, flight altitude) and the date of the study (*i.e.*, crop and weed phenological stage) were also key factors in the accuracy of the classification, mainly in the weed-infested zones (Table 2). According to the results obtained for the weed-infested frames using UAV images captured at 40 m with either of the cameras, the best time for weed detection in early-season sunflower fields was approximately 52 days after sowing (date 2). At this altitude, our results showed that the images captured before this date were more suitable for weed detection than the images captured later in the growing season. However, the best time for weed detection differed for each type of image at higher flight altitudes. If the visible-light camera was used at 60 or 80 m, the best results were obtained for date 3 (57 DAS) because this camera was ineffective on the earliest dates due to the small size of the weed plants and some degree of confusion between their bright leaves and the bare background soil. This problem was minor with the multispectral camera because of the near-infrared information provided in these images, and the results were slightly better on date 1 (44 DAS) than on date 3 at 60, 80 and 100 m.

Considering both weed-infested and weed-free zones, the accuracy obtained with the multispectral camera (by using NDVI images) was 14%, 18% and 5% higher than the accuracy achieved with the visible-light camera (by using ExG images) on dates 1, 2 and 3, respectively. These results have relevant implications for choosing the most appropriate camera because the visible-light camera is a low-cost sensor, whereas the multispectral camera is a costly sensor. Moreover, the visible-light camera generates higher spatial resolution imagery, and its images cover a larger area of study in comparison with the multispectral camera. The errors observed in the ExG images were mainly due to false negative (*i.e.*, no-detection of weeds); the NDVI images detected weed plants in a higher number of frames, but weed coverage was underestimated in some cases. The latter errors could be more acceptable to the farmers because they usually prefer a conservative option and avoid leaving weed patches untreated. Importantly, the OBIA algorithm successfully detected 100% of the crop rows and almost all the weed-free frames in the majority of the cases. This result is essential for providing accurate information to the decision-making system and to help the farmers select, with near 100% confidence, the weed-free areas where the site-specific weed control equipment does not need to go. This is very relevant not only for reducing herbicide applications but for optimising energy (fuel) and field operating time and expense.

**Table 2:** Accuracy assessment of weed detection as affected by camera (vegetation index), date (phenological stage) and flight altitude. In columns, percentage of ground-truth frames correctly and incorrectly classified (see Section 2.4 and Figure 4).

Flight date (Phenological Stage)	Weed Presence	Flight Altitude (m)	Camera (Vegetation Index)							
			Visible-Light (ExG)				Multispectral (NDVI)			
			Correct (%)	Under-Estimated (%)	False − (%)	False + (%)	Correct (%)	Under-estimated (%)	False − (%)	False + (%)
Date 1–44 DAS (4 true leaves)	Weed	40	71	5	14	10	71	10	19	-
		60	43	10	43	4	62	10	28	-
		80	29	10	48	13	57	10	33	-
		100	19	10	24	47	43	14	29	14
	No weed	40	100	-	-	-	100	-	-	-
		60	100	-	-	-	100	-	-	-
		80	44	-	-	56	100	-	-	-
		100	33	-	-	67	100	-	-	-
Date 2–50 DAS (5–6 true leaves)	Weed	40	77	5	14	4	91	9	-	-
		60	64	18	14	4	62	19	19	-
		80	45	14	27	14	55	9	36	-
		100	45	5	9	41	50	14	27	9
	No weed	40	88	-	-	12	100	-	-	-
		60	88	-	-	12	100	-	-	-
		80	63	-	-	37	100	-	-	-
		100	37	-	-	63	88	-	-	12
Date 3–57 DAS (7–8 true leaves)	Weed	40	68	14	18	-	60	7	33	-
		60	68	14	18	-	50	9	36	5
		80	50	-	14	36	41	9	36	14
		100	27	-	41	32	41	-	45	14
	No weed	40	88	-	-	12	100	-	-	-
		60	100	-	-	-	88	-	-	12
		80	63	-	-	37	88	-	-	12
		100	63	-	-	37	100	-	-	-

## 4. Conclusions

Until now, obtaining weed infestation maps early in the growing season has been a great challenge because of the reduced size of the weed and crop plants and their spectral similarity at an early phenological stage. This challenge has been overcome in this investigation by the combined use of an Unmanned Aerial Vehicle (UAV), remote images captured with cameras at visible and near-infrared spectral ranges, and application of object-based image analysis (OBIA) techniques. With both cameras, the highest accuracy in weed detection was achieved with the images captured at 40 m on date 2 (50 days after sowing, DAS) when weeds and sunflower plants had 5–6 true leaves (code 15–16, BBCH scale). On this date, up to 91% accuracy was attained with the images captured by the multispectral camera. At 40 m, the images captured sooner (date 1) reported slightly better results than the images captured later (date 3). However, from 60 m altitude and higher, the images captured with the visible-light camera reported notably better results on date 3 because of the larger size of the weed plants and less confusion distinguishing between crop-row edges and weeds. The source of errors was different for each scenario studied. In general, the errors in the weed-infested zones were mostly attributed to no-detection or underestimation of weeds, whereas the errors in the weed-free zones were due to the wrong classification of the crop-row edges as weeds. This latter type of error was more accentuated in the images captured at higher altitudes due to their lower spatial resolution that blurred spectral detection.

Extrapolating our results to practical use for farmers and prior to performing an UAV flight operation, it is recommended that several factors be considered: (1) camera characteristics and price; (2) area covered by each flight; (3) degree of accuracy needed; and (4) agronomic objective. Therefore, the information reported in this article might be very useful for commercial companies that offer UAV services to farmers or to farmers who own their UAV and must decide on the type of camera (*i.e.*, spatial and spectral sensor resolution) to be used and the optimal flight altitude needed to generate a suitable weed map of the sunflower field early in the season to apply site-specific weed management operations.
